# Does adding ginger extract to a preoperative carbohydrate drink improve outcomes in enhanced recovery after elective neuro-oncologic craniotomy? A randomized controlled trial

**DOI:** 10.3389/fnut.2025.1624176

**Published:** 2025-10-03

**Authors:** Anukoon Kaewborisutsakul, Chanatthee Kitsiripant, Chaitong Churuangsuk, Panupong Puttarak, Onnicha Suntornlohanakul, Khanin Khanungwanitkul, Athithan Rattanaburi, Supattra Uppanisakorn, Kanisorn Sungkaro, Chin Taweesomboonyat, Thara Tunthanathip, Thakul Oearsakul, Sakchai Sae-Heng

**Affiliations:** ^1^Neurological Surgery Unit, Department of Surgery, Faculty of Medicine, Prince of Songkla University, Songkhla, Thailand; ^2^Department of Anesthesiology, Faculty of Medicine, Prince of Songkla University, Songkhla, Thailand; ^3^Clinical Nutrition and Obesity Medicine Unit, Department of Internal Medicine, Faculty of Medicine, Prince of Songkla University, Songkhla, Thailand; ^4^Department of Pharmacognosy and Pharmaceutical Botany, Faculty of Pharmaceutical Sciences, Prince of Songkla University, Songkhla, Thailand; ^5^Phytomedicine and Pharmaceutical Biotechnology Excellence Center, Prince of Songkla University, Songkhla, Thailand; ^6^Endocrinology and Metabolism Unit, Department of Internal Medicine, Faculty of Medicine, Prince of Songkla University, Songkhla, Thailand; ^7^Department of Radiology, Faculty of Medicine, Prince of Songkla University, Songkhla, Thailand; ^8^Division of Gynecologic Oncology, Department of Obstetrics and Gynecology, Faculty of Medicine, Prince of Songkla University, Songkhla, Thailand; ^9^Clinical Research Center, Faculty of Medicine, Prince of Songkla University, Songkhla, Thailand

**Keywords:** ginger extract, gingerol, postoperative nausea and vomiting, enhanced recovery after surgery, preoperative carbohydrate loading, brain tumor

## Abstract

**Introduction:**

Integrating ginger, which is effective in preventing postoperative nausea and vomiting (PONV), into perioperative nutritional strategies for neurosurgical patients may enhance postoperative recovery. In this study, we evaluated whether adding standardized ginger extract to a preoperative carbohydrate drink improves postoperative outcomes in patients undergoing elective neuro-oncologic craniotomy with enhanced recovery after surgery (ERAS).

**Methods:**

This prospective, double-blind, randomized controlled trial enrolled adult patients scheduled for elective neuro-oncologic craniotomy. Participants were randomly assigned to receive either a carbohydrate drink containing standardized ginger extract (ginger group) or an identical carbohydrate drink without it (control group) 2 h before anesthesia induction. All patients received co-treatment following the institutional ERAS protocol. The primary outcomes included the incidence of PONV, nausea severity, vomiting episodes, and rescue antiemetic use within 48 h. Secondary outcomes assessed patient well-being and metabolic and inflammatory responses.

**Results:**

In total, 48 patients were enrolled. The incidence of PONV did not significantly decrease (25% vs. 37.5%; *p* = 0.534), but vomiting episodes were significantly reduced (6 vs. 23 episodes; *p* = 0.003). Moreover, Poisson regression confirmed a lower incidence rate of vomiting in the ginger group than on the control group [incidence rate ratio (IRR): 0.32, 95% CI 0.16–0.80, *p* = 0.017]. No significant differences were found in metabolic markers, inflammatory responses, or well-being scores.

**Discussion:**

Preoperative administration of ginger-enriched carbohydrate drinks effectively reduced the number of vomiting episodes following elective craniotomy. Although other clinical and laboratory outcomes remained unchanged, this nutritional strategy may be beneficial for inclusion in ERAS protocols for elective craniotomy patients.

**Clinical trial registration:**

www.ThaiClinicalTrial.org, identifier TCTR20220124002.

## 1 Introduction

Enhanced recovery after surgery (ERAS) has become a cornerstone in perioperative care, aiming to minimize surgical stress, accelerate functional recovery, and improve patient outcomes (1). Although ERAS protocols have been extensively adopted across various surgical subspecialties, their role in neurosurgery, particularly in elective neuro-oncologic craniotomy, remains under investigation. Craniotomy imposes substantial physiological stress, including metabolic alterations, insulin resistance, and catabolic states, which contribute to postoperative fatigue, nausea, and prolonged hospitalization (2, 3). As essential components of the non-neurosurgical ERAS protocol, nutritional assessment and intervention are increasingly recognized for their potential to optimize recovery following elective craniotomy (4–6).

Preoperative carbohydrate loading has emerged as a key component of ERAS, designed to counteract perioperative catabolic stress and improve metabolic recovery (7). Unlike traditional fasting protocols, carbohydrate drinks reduce postoperative insulin resistance, promote anabolic metabolism, and enhance subjective well-being by alleviating thirst and preoperative anxiety (8). In non-neurosurgical populations, carbohydrate loading has been associated with shorter hospital stays without increasing postoperative complications (9). Randomized trials, such as that by Singh et al. (10) have demonstrated improved immediate postoperative outcomes, including reduced nausea, vomiting, and pain after laparoscopic cholecystectomy. However, the effect of preoperative carbohydrate supplementation in neurosurgical patients remains insufficiently explored, highlighting the need for further investigation (11).

Ginger, a natural antiemetic, has proven effective in reducing nausea and vomiting through multiple pharmacological mechanisms (12). Its active compound, gingerol, modulates gastric motility, inhibits inflammatory mediators, and exerts antagonistic effects on serotonin receptors, contributing to its antiemetic properties (13). Clinical trials in non-neurosurgical populations have established its effectiveness in preventing postoperative nausea and vomiting (PONV), with randomized studies showing approximately a 25–30% reduction in incidence after limb surgery (14) and significant improvements in nausea severity and vomiting frequency when ginger extract was added to perioperative caloric drinks (15). A recent meta-analysis further confirmed a pooled protective effect of ginger against PONV across diverse surgical settings (16). Considering that up to 70% of neurosurgical patients experience PONV (17, 18), integrating ginger into perioperative nutritional strategies may enhance postoperative recovery (19).

In this study, we aimed to evaluate the efficacy of a novel preoperative carbohydrate drink containing standardized ginger extract in patients undergoing elective neuro-oncologic craniotomy. We hypothesized that adding ginger to a carbohydrate drink will provide superior benefits in reducing PONV, improving metabolic response, and enhancing patient well-being compared with standard preoperative carbohydrate loading alone.

## 2 Materials and methods

### 2.1 Study design and setting

This study was a prospective, double-blind, randomized controlled trial conducted at a university hospital in Southern Thailand between January 2023 and June 2024. Patients undergoing elective neuro-oncologic craniotomy were recruited, and written informed consent was obtained prior to enrollment. The Institutional Ethical Board Committee (REC.65-075-10-1) approved the study conducted by the Declaration of Helsinki and Good Clinical Practice guidelines. This trial was registered at www.ThaiClinicalTrial.org (Trial ID: TCTR20220124002).

### 2.2 Patient selection

Eligible participants were adults aged ≥ 18 years with a confirmed diagnosis of a single supratentorial intracranial tumor as confirmed by preoperative neuroimaging and scheduled for elective craniotomy. To ensure adequate baseline functional status, patients were required to have an American Society of Anesthesiologists (ASA) physical status classification of I–III and a Karnofsky Performance Score (KPS) ≥ 80.

Exclusion criteria comprised emergency craniotomy, known allergy to ginger or its extract, pregnancy, and severe hepatic or renal dysfunction. Additional exclusions encompassed preoperative steroid or immunosuppressant use within 7 days, cognitive impairment, diabetes mellitus, and a body mass index (BMI) outside the range of 20 to 30 kg/m^2^. Patients with gastrointestinal disorders, including gastroesophageal reflux disease (GERD) or bowel obstruction, were also excluded.

### 2.3 Sample size calculation

The sample size was calculated based on a prior study by Rizvanović et al. (20) which investigated the impact of preoperative oral carbohydrate loading on postoperative outcomes. Assuming an 80% power and a two-sided alpha of 0.05, a minimum of 22 participants per group was required to detect a clinically meaningful difference in nausea incidence. To account for possible attrition, the final target enrollment was 24 participants per group (48 total). All randomized patients completed the study and were included in the final analysis.

### 2.4 Randomization and blinding

Participants were randomly assigned in a 1:1 ratio to either the experimental ginger-carbohydrate (ginger) or the placebo (control) groups using a computer-generated block of four randomization. An independent researcher generated the allocation sequence and ensured allocation concealment.

This was a double-blind study in which patients and outcome assessors were blinded to group assignments. Blinding was maintained by ensuring that the intervention and placebo drinks were identical in color, taste, and packaging.

### 2.5 Intervention

Patients in the ginger group received 400 mL of a novel preoperative carbohydrate drink, formulated and manufactured in accordance with Good Manufacturing Practice standards. The formulation contains complex carbohydrates (50 grams total, primarily maltodextrin and isomalt) and standardized ginger extract prepared using a microwave extraction and spray-drying process. The content of the active compound 6-gingerol was verified by high-performance liquid chromatography (HPLC), yielding 10 mg of 6-gingerol per serving. The selected dose was based on ranges reported in previous systematic reviews (21, 22). Microbiological and heavy metal analyses confirmed compliance with international safety standards.

The control group received an identical carbohydrate drink in terms of volume, calories (200 calories per serving), and osmolality but without active ginger compounds. Instead, it contained an artificial ginger flavor to maintain blinding. Both drinks were prepared by a certified researcher, who was not involved in patient care, and were administered 2 h before anesthesia induction.

### 2.6 Perioperative management

A standardized anesthetic and surgical protocol were implemented for all procedures. All craniotomies were performed by board-certified neurosurgeons utilizing neuronavigation and intraoperative neurophysiological monitoring as required. Perioperative management adhered to the ERAS principles, following protocols established in previous studies (20, 23, 24). However, the PONV prophylaxis component was intentionally withheld to isolate and quantify the antiemetic efficacy of ginger supplementation. Patient safety was ensured through a predefined rescue protocol: metoclopramide (10 mg intravenously) was administered as first-line therapy for nausea scores ≥ 4 on the visual analog scale (VAS) or for any vomiting episode, with repeat doses every 8 h as needed. Ondansetron (8 mg intravenously, repeatable every 8 h) was used as second-line therapy for persistent symptoms. All rescue medication decisions were made by blinded clinicians following these predetermined protocols to minimize bias.

### 2.7 Outcome measures

The primary outcome was the incidence of PONV within 48 h after surgery, including the severity of nausea assessed using the VAS, total number of vomiting episodes, and use of rescue antiemetics. Nausea severity was categorized as none, mild, moderate, or severe. Secondary outcomes included patient-reported well-being scores, such as thirst, hunger, anxiety, fatigue, and dry mouth, as applied in previous ERAS trials (20). Metabolic and inflammatory responses [glucose, insulin, cortisol, C-reactive protein (CRP), interleukin-6 (IL-6), serum albumin, and urinary nitrogen balance] were chosen according to perioperative physiology literature (25). Insulin resistance was assessed using the Homeostasis Model Assessment of Insulin Resistance (HOMA-IR) as described in previous ERAS-related trials (20). Fasting blood glucose was measured using an automated chemistry analyzer with the enzymatic (hexokinase) method. Serum insulin and cortisol were measured using electrochemiluminescence immunoassay (ECLIA). CRP was determined by immunoturbidimetric assay, and IL-6 was measured by chemiluminescent immunoassay (CLIA). Urinary nitrogen balance was determined using the enzymatic urease method.

### 2.8 Statistical analysis

All statistical analyses were conducted using an intention-to-treat approach. Comparisons between groups were performed using the independent t-test or Mann–Whitney U test for continuous variables and Fisher’s exact test or the chi-squared test for categorical variables. A Poisson regression model was used to analyze the incidence rate of vomiting episodes while adjusting for intraoperative confounders, including operative time and anesthesia duration. A *p*-value of < 0.05 was considered statistically significant.

## 3 Results

**Figure 1:**
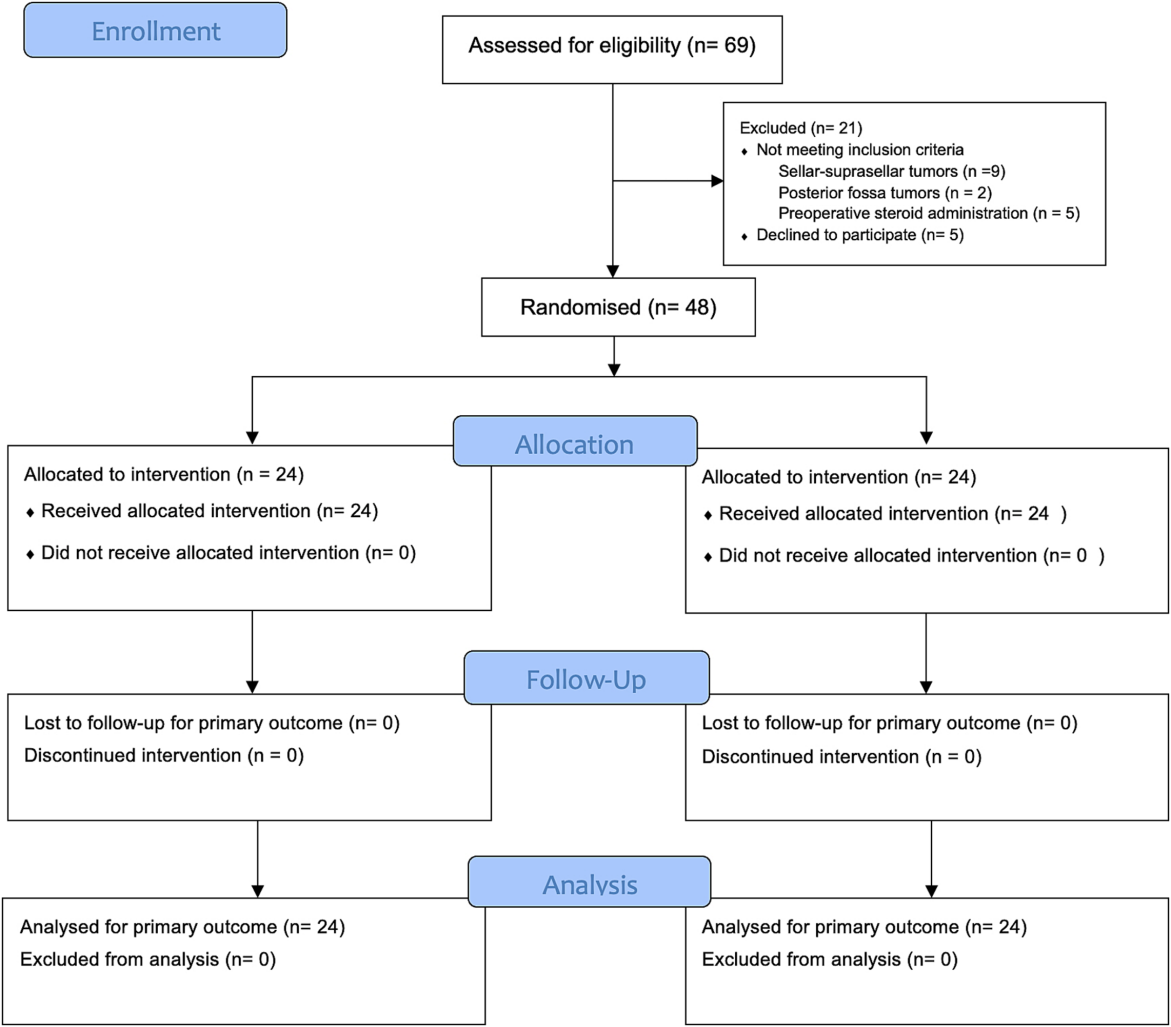
CONSORT 2025 flow diagram.

Of 69 patients screened for eligibility, 21 patients were excluded: 16 who did not meet the inclusion criteria—9 with sellar-suprasellar tumors, 2 with posterior fossa tumors, and 5 who had received preoperative steroids—and 5 who declined to participate. Finally, 48 patients were enrolled and evenly assigned to the ginger (24 patients) and control (24 patients) groups. Baseline demographic and clinical characteristics were generally well-balanced between the two groups. The flow of participants throughout the trial is illustrated in Figure 1, in accordance with the CONSORT 2025 guidelines (26).

No significant differences were observed in age, BMI, ASA classification, KPS, Apfel PONV score, tumor type, tumor location, or comorbid conditions, such as hypertension, dyslipidemia, and anemia. However, the operative time was significantly longer in the ginger group than in the control group (417.83 ± 173.19 min vs. 316.04 ± 169.04 min, *p* = 0.015). Similarly, the anesthesia time was significantly longer in the ginger group (495.21 ± 179.05 min vs. 399.04 ± 188.14 min, *p* = 0.030), as shown in Table 1.

**Table 1:** Patients’ characteristics.

Characteristics	Ginger group	Control group	*p* value^a^
	Patients, *n* (%)	Patients, *n* (%)	
Total number	24	24	
Age, year, (mean ± SD)	46.21 ± 14.07	49.21 ± 12.29	0.435
Female	19 (79.1)	12 (50)	0.069
BMI, kg/m^2^, (mean ± SD)	26.54 ± 5.27	26.15 ± 4.57	0.613
ASA class			0.234
II	24 (100)	21 (87.5)	
III	0	3 (12.5)	
KPS			0.200
80	0	3 (12.5)	
90	22 (91.7)	19 (79.2)	
100	2 (8.3)	2 (8.3)	
Non-smoker status	23 (95.8)	19 (79.2)	0.188
Postoperative opioids	24 (100)	24 (100)	1.000
Apfel PONV score			0.191
1	1 (4.2)	4 (16.7)	
2	4 (16.7)	8 (33.3)	
3	17 (70.8)	11 (45.8)	
4	2 (8.3)	1 (4.2)	
Type of tumors			1.000
Meningioma	14	15	
Low grade glioma	4	2	
High grade glioma	2	5	
Metastasis	4	1	
Others	0	1	
Location of tumors			1.000
Intra-parenchymal	10	9	
Extra-parenchymal	14	15	
Preexisting diseases			
Hypertension	4 (16.7)	4 (16.7)	1.000
Dyslipidemia	5 (20.8)	5 (20.8)	1.000
Anemia	7 (29.2)	2 (8.3)	0.137
Operative profiles			
Operative time, mins (mean ± SD)	417.83 ± 173.19	316.04 ± 169.04	0.015
Anesthesia time, mins (mean ± SD)	495.21 ± 179.05	399.04 ± 188.14	0.030
Estimated blood loss, mL (mean ± SD)	847.92 ± 484.43	669.58 ± 686.13	0.051

^a^All means and SD were compared with Mann–Whitney U statistic (except age). ASA, American Society of Anesthesiologists physical status classification; BMI, body mass index; KPS, Karnofsky Performance Status; mins, minutes; mL, milliliter; PONV, postoperative nausea and vomiting; SD, standard deviation.

### 3.1 Primary outcome

At 48 h postoperatively, the incidence of PONV did not differ significantly between the ginger (25%) and control (37.5%) groups (*p* = 0.534) (Table 2). In the ginger group, 18 patients reported no or mild nausea, whereas 15 patients in the control group fell into these categories. Moderate nausea was observed in six and four patients in the ginger and control groups, respectively, whereas no cases of severe nausea were reported in either group. The difference in nausea severity between groups was not statistically significant (*p* = 0.164). The total number of vomiting episodes was significantly lower in the ginger group than in the control group (6 vs. 23 episodes, *p* = 0.003). Vomiting was observed in four patients in the ginger group and eight patients in the control group. The use of antiemetic medication was comparable between groups. Metoclopramide was administered to 14 and 20 patients in the ginger and control groups, respectively (*p* = 0.770). Although the mean total dose of metoclopramide was lower in the ginger group (11.3 ± 12.9 mg) than in the control group (42.5 ± 60.0 mg), the difference was not statistically significant (*p* = 0.110). The use of ondansetron was minimal, with two patients receiving it in the ginger group and four in the control group (*p* = 1.000).

**Table 2:** Postoperative nausea and vomiting outcomes at 48 h.

Outcomes	Ginger group	Control group	*p* value
	(*n* = 24)	(*n* = 24)	
PONV, *n* (%)	6 (25)	9 (37.5)	0.534
Nausea severity, *n* (none/mild/moderate/severe)	18/6/0/0	15/5/4/0	0.164
Total vomiting event	6 (4 patients)	23 (8 patients)	0.003
Rescue antiemetic used			
Metoclopramide, *n*	14	20	0.770
Dose of metoclopramide, mg, (Mean ± SD)	11.3 ± 12.9	42.5 ± 60.0	0.110
Ondansetron, *n*	2	4	1.000

mg, milligram; PONV, postoperative nausea and vomiting; SD, standard deviation.

Poisson regression analysis, adjusting for operative and anesthesia times, confirmed that the ginger group had a significantly lower incidence rate of vomiting events (incidence rate ratio [IRR]: 0.32, 95% CI 0.16–0.80, *p* = 0.017) than that of the control group. Neither operative time (*p* = 0.741) nor anesthesia duration (*p* = 0.702) significantly influenced vomiting events. In a sensitivity analysis including baseline CRP as an additional covariate, the ginger group remained protective against vomiting (IRR 0.53, 95% CI 0.16–1.77, *p* = 0.299), although the association was no longer statistically significant. Baseline CRP alone was not a significant predictor of vomiting (IRR 0.69, 95% CI 0.43–1.10, *p* = 0.121).

### 3.2 Secondary outcome

**Figure 2:**
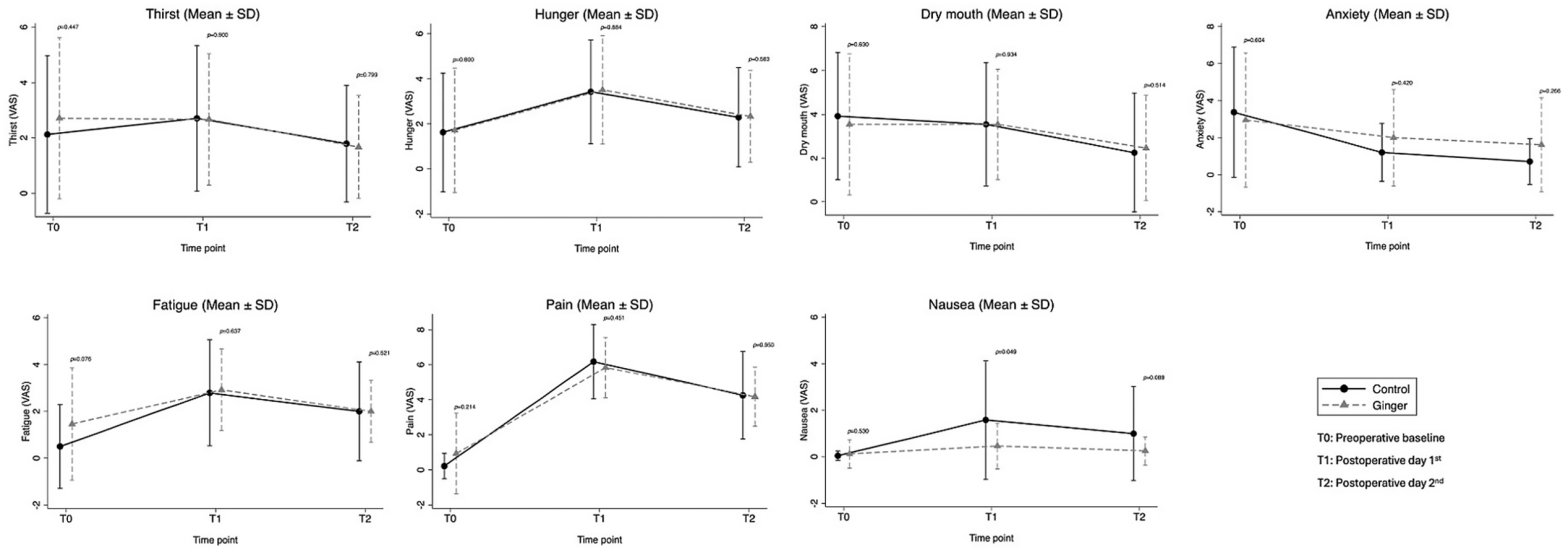
Patient-reported outcomes (VAS) at baseline (T0), postoperative day 1 (T1), and day 2 (T2). Nausea severity was lower in the ginger group on postoperative day 1 (*p* = 0.049), with no significant differences for other parameters.

Patient-reported well-being outcomes, including thirst, hunger, dry mouth, anxiety, fatigue, pain, and nausea, were assessed using VAS at three time points: preoperatively (T0), postoperative day 1 (T1), and postoperative day 2 (T2) (Figure 2; Supplementary Table 1). No significant differences were found between the ginger and control groups for thirst, hunger, dry mouth, anxiety, fatigue, or pain at any time point. For nausea severity, baseline VAS scores were similar between groups (*p* = 0.530). However, on T1, nausea severity was significantly lower in the ginger group than in the control group (0.46 ± 0.98 vs. 1.58 ± 2.55, *p* = 0.0495). By T2, nausea severity remained lower in the ginger group than in the control group (0.25 ± 0.61 vs. 1.00 ± 2.02); however, the difference was not statistically significant (*p* = 0.088).

No significant differences were observed between the ginger and control groups in term of glucose, insulin, HOMA-IR, cortisol, IL-6, or albumin levels at any time point (Table 3). By contrast, preoperative CRP levels were significantly higher in the ginger group (6.00 ± 8.20 mg/L) than in the control group (2.24 ± 3.93 mg/L, *p* = 0.001). However, the postoperative levels were similar (*p* = 0.529 and *p* = 0.893 on T1 vs. T2, respectively). Further details on how baseline CRP was addressed in relation to vomiting outcomes are presented in Section 3.1.

**Table 3:** Comparison of metabolic and inflammatory response markers at three time points.

Variables	Time	Ginger group (*n* = 24)	Control group (*n* = 24)	*p* value
		Mean ± SD	Mean ± SD	
Glucose (mg/dL)	T0	96.42 ± 16.88	92.92 ± 9.58	0.687
	T1	131.50 ± 35.98	129.33 ± 29.39	0.960
	T2	114.38 ± 31.07	101.92 ± 26.78	0.3693
Insulin (μU/mL)	T0	13.02 ± 6.43	11.68 ± 5.74	0.621
	T1	14.10 ± 9.27	11.59 ± 5.69	0.797
	T2	10.69 ± 7.27	10.33 ± 7.82	0.926
HOMA-IR	T0	3.13 ± 1.65	2.73 ± 1.46	0.370
	T1	4.80 ± 3.62	3.87 ± 2.43	0.845
	T2	3.27 ± 2.84	2.83 ± 2.55	0.749
Cortisol (μg/dL)	T0	13.61 ± 7.10	13.05 ± 4.77	0.510
	T1	41.29 ± 75.13	20.16 ± 7.78	0.650
	T2	18.16 ± 6.67	16.91 ± 5.74	0.205
CRP (mg/L)	T0	6.00 ± 8.20	2.24 ± 3.93	0.001^*^
	T1	29.21 ± 16.08	28.33 ± 21.37	0.529
	T2	110.48 ± 61.61	107.78 ± 64.19	0.893
IL-6 (pg/mL)	T0	7.25 ± 6.43	4.41 ± 1.91	0.080
	T1	53.15 ± 50.65	67.77 ± 55.21	0.140
	T2	42.75 ± 25.74	48.47 ± 37.68	0.688
Albumin (g/dL)	T0	4.03 ± 0.39	4.10 ± 0.38	0.320
	T1	3.37 ± 3.57	3.57 ± 0.41	0.060
	T2	3.33 ± 0.23	3.45 ± 0.35	0.080
Urine nitrogen (mmol/day)	T0	650.74 ± 296.47	630.07 ± 345.6	0.840
	T1	245.4 ± 155.35	272.29 ± 167.72	0.490
	T2	311.78 ± 213.59	404.38 ± 189.94	0.042^*^

^*^Statistically significant (*p* < 0.05). SD, standard deviation; T0, Preoperative baseline; T1, Postoperative day 1; T2, Postoperative day 2.

Urinary nitrogen levels showed no significant differences at T0 (*p* = 0.840) or on T1 (*p* = 0.490). However, by T2, the ginger group had significantly lower levels than the control group did (311.78 ± 213.59 vs. 404.38 ± 189.94, *p* = 0.042).

The total length of hospital stay was 10.6 ± 1.7 days, with no significant difference between the ginger (10.7 ± 0.16 days) and control (10.5 ± 2.57 days) groups (*p* = 0.958). Similarly, the total hospital cost was 205,071.9 ± 14,595.7 baht. Although the ginger group had a higher mean hospital cost (220,814.6 ± 20,499.7 baht) than that in the control group (189,329.2 ± 20,709.2 baht), this difference was not statistically significant (*p* = 0.286). No mortality, reoperation, or hospital readmission cases were recorded within 30 days in either group.

## 4 Discussion

This study evaluated the effect of integrating ginger into a preoperative carbohydrate drink within an ERAS protocol for elective neuro-oncologic craniotomy. The results demonstrated that ginger supplementation reduced postoperative vomiting episodes by 68% (IRR 0.32, 95% CI: 0.16–0.80), emphasizing its potential role in improving perioperative care for neuro-oncologic patients. Although the reduction in vomiting episodes was statistically significant, its clinical significance is also notable, as fewer vomiting events after craniotomy may improve patient comfort, decrease the risk of elevated intracranial pressure, and reduce wound-related complications. However, no significant differences between the ginger and control groups were observed in metabolic responses, inflammatory markers, or other clinical outcomes. These negative secondary findings may reflect the limited sample size, baseline variability between groups (e.g., higher preoperative CRP in the ginger group), and the possibility that ginger’s primary effect is restricted to the emetic pathway rather than broader systemic metabolic regulation.

PONV is a significant concern after craniotomy, with an incidence rate of 16–70% in patients without perioperative prophylaxis (17, 27, 28). It involves multiple physiological pathways, including the vestibular system, chemoreceptor trigger zone, and gastrointestinal mechanisms. Major risk factors include volatile anesthetics, opioids, female sex, motion sickness history, and non-smoking status. Consequently, the American Society of Enhanced Recovery and Society for Ambulatory Anesthesia guidelines recommend a multimodal prevention strategy that includes risk stratification, pharmacological prophylaxis, and rescue therapy (19). Additionally, uncontrolled PONV can lead to dehydration, electrolyte imbalances, delayed recovery, and complications, including increased intracranial pressure and wound dehiscence (17).

This trial aligns with existing guidelines by incorporating preoperative carbohydrate loading and ginger supplementation into a multimodal strategy for PONV prophylaxis. Carbohydrate drinks have been shown to mitigate the metabolic stress and catabolic effects of preoperative fasting (29). Concomitantly, ginger acts through multiple mechanisms, including serotonin and dopamine receptor antagonism, enhancement of gastric motility, and possible anti-inflammatory effects (12, 13). The combination of these nutritional interventions significantly reduced vomiting episodes, highlighting their potential as adjuncts to PONV prophylaxis protocols in neurosurgical patients (30).

Multiple studies in abdominal, gynecologic, and orthopedic procedures have demonstrated that ginger supplementation can reduce the incidence and severity of PONV (14, 31, 32). In a randomized controlled trial, Sihombing et al. (15) reported a 30% reduction in postoperative nausea following the addition of ginger extract to a preoperative caloric beverage in patients undergoing elective surgery. This effect is comparable to the significant reduction in vomiting episodes observed in the present study. Although evidence on ginger supplementation in neurosurgical populations remains limited, our findings bridge this knowledge gap by evaluating a combined nutritional and antiemetic strategy tailored to the perioperative needs of this high-risk group.

The observed reduction in vomiting episodes in the ginger group likely stems from ginger’s multiple mechanisms of action. Gingerol, the primary active component of ginger, acts as a 5-HT3 receptor antagonist, similar to ondansetron, effectively blocking serotonin-mediated nausea and vomiting pathways. Additionally, it modulates gastric motility by enhancing gastric emptying and reducing gastric dysrhythmias, which may mitigate the gastric stasis commonly observed after surgery. The anti-inflammatory properties of ginger, including inhibition of prostaglandin synthesis and reduction of pro-inflammatory cytokines, may further contribute to its antiemetic effects (13, 16, 33). Interestingly, our study found greater efficacy in reducing vomiting events than overall PONV incidence, suggesting that ginger may preferentially target the emetic reflex pathway while having a more modest effect on the subjective sensation of nausea. Collectively, these findings provide both mechanistic plausibility and clinical evidence supporting ginger supplementation as a cost-effective, widely available adjunct to multimodal ERAS antiemetic protocols, with particular value for neurosurgical patients at high risk of PONV. From a clinical perspective, standardized ginger extract is inexpensive and globally accessible, making it a practical option even in resource-limited settings. The safety of our formulation has also been demonstrated in a recent pilot randomized crossover trial assessing gastric emptying, glycemic responses, and fasting discomfort (34), supporting its use alongside conventional prophylactic strategies. Thus, integrating ginger-enriched carbohydrate drinks into ERAS pathways is both feasible and safe for widespread application.

Postoperative inflammatory and metabolic responses were assessed using multiple laboratory markers. Although preoperative CRP levels were significantly higher in the ginger group than in the control group, postoperative CRP levels were comparable on both T1 and T2. This suggests that while baseline differences existed, the postoperative trajectories were similar across groups, highlighting that the decline in CRP likely reflects the natural resolution of surgical inflammation rather than a ginger-specific anti-inflammatory effect. Although prior experimental studies have demonstrated the anti-inflammatory properties of ginger—primarily through cytokine modulation and reduction of oxidative stress—these clinical findings do not provide direct evidence supporting a significant role for ginger in attenuating postoperative systemic inflammation in this surgical setting (13).

Protein catabolism is a typical physiological response to major surgery, including craniotomy, and can be quantified using various methods, such as postoperative urinary nitrogen measurements. Liu et al. (35) demonstrated that preoperative carbohydrate loading significantly reduced negative nitrogen balance in patients undergoing elective craniotomy. In the present study, urinary nitrogen levels were significantly lower in the ginger group than in the control group on T2. However, this finding was not accompanied by consistent changes in other metabolic indicators, such as glucose, insulin, or albumin, and may therefore represent an incidental observation. Moreover, urinary nitrogen measurements can be influenced by perioperative factors, including fluid balance, renal function, and dietary intake, which were not controlled in this study. For these reasons, the reduction in urinary nitrogen should be interpreted with caution. Further research with larger sample sizes and more comprehensive metabolic assessments is warranted before concluding that ginger has a clinically meaningful effect on perioperative protein preservation.

This study has several limitations that should be considered. Although the sample size was adequate to detect a significant difference in vomiting episodes, it may have been underpowered to identify differences in overall PONV incidence and the effects of the intervention on metabolic and inflammatory markers. As this was a single-center trial, the generalizability of the findings to broader patient populations and clinical settings is limited. Despite randomization, imbalances in baseline characteristics were observed, particularly in operative and anesthetic times, which could have introduced confounding despite statistical adjustment. Furthermore, the heterogeneity of tumor types and locations among participants may have contributed to variability in PONV risk. In addition, the strict inclusion and exclusion criteria applied in this trial, such as limiting BMI and excluding patients with diabetes, may further limit the generalizability of our findings. These measures were initially implemented to ensure safety in patients, particularly for those at risk of delayed gastric emptying. Although this conservative approach limited external validity, subsequent work by our group has demonstrated the safety of the novel carbohydrate drink in a broader population, including patients at risk of impaired gastric emptying (34). Finally, this study was not explicitly designed to evaluate hospital length of stay or total hospital cost; these outcomes were included for exploratory analysis only.

Future research should address these limitations through larger, multicenter trials with more homogeneous patient populations. Investigating the efficacy of ginger-containing carbohydrate drinks in various neurosurgical procedures, such as posterior fossa surgeries—which carry an even higher risk of PONV—would further validate the generalizability of these findings. Dose-finding studies are also warranted to determine the optimal concentration of ginger extract that balances maximal antiemetic efficacy with safety. In addition, further exploration of ginger’s effects on metabolic and inflammatory responses, particularly with extended follow-up periods, may reveal benefits beyond PONV reduction. From a broader perspective, this intervention could also be evaluated in other high-risk surgical populations outside neurosurgery, such as abdominal or thoracic surgery, where PONV and catabolic stress are similarly significant concerns.

## 5 Conclusion

The addition of standardized ginger extract to a preoperative carbohydrate drink significantly reduced postoperative vomiting episodes in patients undergoing elective neuro-oncologic craniotomy. Although ginger supplementation did not significantly affect overall PONV incidence or metabolic and inflammatory parameters, the observed reduction in vomiting represents a clinically meaningful benefit that remained significant after adjustment for confounding factors. These results support the incorporation of ginger-enriched carbohydrate drinks as a simple, and effective adjunct to enhanced recovery protocols in neurosurgical patients, potentially improving postoperative outcomes.

## Data Availability

The original contributions presented in the study are included in the article/Supplementary material, further inquiries can be directed to the corresponding author.
