# Supplementary material for: Does adding ginger extract to a preoperative carbohydrate drink improve outcomes in enhanced recovery after elective neuro-oncologic craniotomy? A randomized controlled trial
Source: Front Nutr. 2025 Oct 3;12:1624176. doi: 10.3389/fnut.2025.1624176 (PMC12532379; doi:10.3389/fnut.2025.1624176)
Supplement: Supplementary file 1 [file Table_1.docx]

**Supplementary Table 1.** Comparison of patient-reported well-being outcomes using the visual analog scale among three time points

|  | Time | Ginger group (n = 24) | Control group (n = 24) | *p* value |
| --- | --- | --- | --- | --- |
|  |  | **Mean ± SD** | **Mean ± SD** |  |
| Thirst | T0 | 2.71 ± 2.91 | 2.12 ± 2.85 | 0.447 |
|  | T1 | 2.67 ± 2.37 | 2.71 ± 2.63 | 0.900 |
|  | T2 | 1.67 ± 1.86 | 1.79 ± 2.11 | 0.799 |
| Hunger | T0 | 1.71 ± 2.76 | 1.62 ± 2.63 | 0.800 |
|  | T1 | 3.50 ± 2.40 | 3.42 ± 2.30 | 0.884 |
|  | T2 | 2.33 ± 2.04 | 2.29 ± 2.20 | 0.583 |
| Dry mouth | T0 | 3.54 ± 3.22 | 3.92 ± 2.90 | 0.630 |
|  | T1 | 3.54 ± 2.52 | 3.54 ± 2.81 | 0.934 |
|  | T2 | 2.46 ± 2.40 | 2.25 ± 2.71 | 0.514 |
| Anxiety | T0 | 2.96 ± 3.62 | 3.38 ± 3.52 | 0.604 |
|  | T1 | 2.00 ± 2.60 | 1.21 ± 1.56 | 0.420 |
|  | T2 | 1.62 ± 2.53 | 0.71 ± 1.23 | 0.266 |
| Fatigue | T0 | 1.46 ± 2.40 | 0.50 ± 1.79 | 0.076 |
|  | T1 | 2.92 ± 1.74 | 2.79 ± 2.26 | 0.637 |
|  | T2 | 2.00 ± 1.32 | 2.00 ± 2.11 | 0.521 |
| Pain | T0 | 0.92 ± 2.30 | 0.21 ± 0.72 | 0.214 |
|  | T1 | 5.83 ± 1.71 | 6.17 ± 2.12 | 0.451 |
|  | T2 | 4.17 ± 1.69 | 4.25 ± 2.49 | 0.950 |
| Nausea | T0 | 0.12 ± 0.61 | 0.04 ± 0.20 | 0.530 |
|  | T1 | 0.46 ± 0.98 | 1.58 ± 2.55 | 0.049^*^ |
|  | T2 | 0.25 ± 0.61 | 1.00 ± 2.02 | 0.088 |

* Statistically significant (*p* < 0.05*).* SD: standard deviation; T0: Preoperative baseline; T1: Postoperative day 1; T2: Postoperative day 2
